# Clinical efficacy and safety of subtotal resection of adenomyotic lesions based on the Kishi classification: a retrospective case series study

**DOI:** 10.3389/fmed.2026.1831932

**Published:** 2026-06-11

**Authors:** Zhenyue Qin, Dan Song, Zhiyong Dong, Bingying Lu, Weiwei Wei, Jiming Chen

**Affiliations:** 1 Shaoxing Maternity and Child Health Care Hospital, Shaoxing University, Shaoxing, China; 2 Maternity and Child Health Care Affiliated Hospital, Shaoxing University, Shaoxing, China; 3 The Second Affiliated Hospital of Chongqing Medical University, Chongqing, China; 4 The Third Affiliated Hospital of Nanjing Medical University (The Second People’s Hospital of Changzhou), Changzhou, China

**Keywords:** dysmenorrhea, gonadotropin-releasing hormone agonist, Kishi classification, Levonorgestrel-releasing intrauterine system, severe adenomyosis, subtotal resection of adenomyotic lesions

## Abstract

**Background and objective:**

Diagnosis and treatment strategies for adenomyosis are influenced by multiple factors, including patient preferences, adenomyosis subtype, and disease progression, with no standardized protocol currently established. In 2012, Kishi proposed four subtypes of adenomyosis based on magnetic resonance imaging (MRI) to guide clinical treatment. This study focuses on patients with severe adenomyosis who do not desire future fertility but wish to preserve their uterus. It investigates the clinical efficacy of subtotal adenomyotic lesion resection based on the Kishi classification, combined with sequential levonorgestrel-releasing intrauterine system (LNG-IUS) and gonadotropin-releasing hormone agonist (GnRH-a) therapy, in the management of severe adenomyosis, and evaluates the clinical outcomes and safety of this approach.

**Methods:**

We used a descriptive case series design. A retrospective analysis was performed on 34 patients with severe adenomyosis who underwent this treatment protocol at the Department of Gynecology, the Changzhou Second People’s Hospital Affiliated to Nanjing Medical University from December 2017 to October 2022. Clinical parameters, including hemoglobin concentrations, dysmenorrhea severity scores, and menstrual blood loss scores, before and after treatment, were observed and recorded.

**Results:**

All 34 patients successfully underwent surgery. Comparisons of postoperative and preoperative values showed significant improvements in dysmenorrhea severity score, menstrual blood loss score, hemoglobin concentration, serum cancer antigen-125 (CA125) level, and uterine volume (all *p* < 0.001). Only one patient experienced downward displacement of the intrauterine device, and a transvaginal ultrasonography revealed that the upper end of the device was approximately 1.5 cm away from the uterine fundus. One patient exhibited a mild elevation in CA125, accompanied by an increase in dysmenorrhea severity score to 3 points, without a significant increase in menstrual blood loss being observed. No disease progression was noted in any patient.

**Conclusion:**

In patients with severe adenomyosis who do not desire future fertility, who have a strong preference for uterine preservation, and who have failed multiple conservative treatments, this combined regimen was associated with significant improvements in dysmenorrhea severity, menstrual blood loss, anemia, CA125 levels, and uterine volume over a 12-month observation period, without any serious adverse events. This finding suggests that the regimen may be a safe and effective treatment option. However, its exact efficacy still requires validation through prospective controlled studies.

## 1 Introduction

Adenomyosis (AM) is a pathological condition resulting from the invasion and proliferation of endometrial tissue within the myometrium (1). Advanced age has been identified as a risk factor for AM (2, 3), whereas its prevalence is also progressively increasing among young women of reproductive age (4, 5). Although AM is a benign disease, it exhibits biological characteristics similar to those of malignant conditions, including infiltrative growth into adjacent myometrial tissues, rapid proliferation, and even the potential for implantation metastasis. Consequently, its diagnosis and treatment strategies remain a focal point of research. Diagnosis and treatment strategies are influenced by multiple factors, including patient preferences, adenomyosis subtype, and the extent of the lesions. Pharmacological treatments are often ineffective for adenomyosis, making surgical intervention one of the primary treatment approaches. While hysterectomy, a radical procedure, may be difficult for some patients to accept, lesion resection presents a viable treatment option for AM (6). However, there is currently no standardized surgical protocol or procedure for lesion resection, and complete excision of lesions during surgery is often challenging. Owing to the infiltrative nature of AM, postoperative recurrence is highly likely, underscoring the equal importance of postoperative maintenance therapy alongside surgical treatment (7).

In 2012, Kishi et al. (8) proposed a classification of adenomyosis based on MRI, which has provided significant guidance for the clinical diagnosis and treatment of adenomyosis. Given the complexity of adenomyosis treatment, our team explored strategies to achieve better long-term therapeutic outcomes while preserving the uterus in patients who respond poorly to other conservative therapies yet strongly desire uterine preservation. Based on the Kishi classification, we designed this treatment protocol, which improves upon the traditional “double-flap” technique by thoroughly excising adenomyotic lesions and reshaping the resected uterine cavity. Postoperatively, LNG-IUS and GnRH-a were sequentially administered to consolidate efficacy and reduce recurrence rates. This approach has yielded satisfactory clinical outcomes, as detailed below.

## 2 Data and methods

### 2.1 General data

A descriptive case series design was adopted to retrospectively collect data from 34 patients treated for severe adenomyosis in the Department of Gynecology at the Changzhou Second People’s Hospital Affiliated to Nanjing Medical University between December 2017 and October 2022. The inclusion criteria for patients were as follows: (1) severe adenomyosis: according to the International Federation of Gynecology and Obstetrics (FIGO) criteria, cases with lesions involving more than 50% of the myometrial volume were classified as severe, with postoperative pathology confirming adenomyosis (9, 10). All patients underwent preoperative MRI or transvaginal ultrasound (23 patients underwent pelvic MRI, whereas 11 underwent transvaginal ultrasound only). The lesion distribution patterns were classified independently by two attending physicians or physicians of higher rank based on the Kishi classification criteria to guide surgical planning (8); (2) no desire for future fertility but a strong desire to preserve the uterus; (3) failure of conservative medical treatment; and (4) voluntary acceptance of the surgical procedure and provision of signed informed consent. The exclusion criteria for patients were as follows: (1) primary dysmenorrhea rather than secondary dysmenorrhea; (2) suspected concurrent endometrial or cervical lesions based on preoperative diagnostic curettage, cervical examination, hysteroscopy, or other auxiliary tests; and (3) unwillingness to receive postoperative treatment with GnRH-a or being lost to follow-up.

A preliminary assessment of the safety, efficacy, and therapeutic outcomes of the modified surgical technique in this study was conducted by analyzing and comparing various outcome measures before and after treatment in 34 patients. The treatment protocol involved performing a modified subtotal resection of adenomyotic lesions during surgery, intraoperative placement of the LNG-IUS (Mirena), followed by sequential GnRH-a therapy postoperatively. Among the 34 patients, the median age of patients was 43 years (range: 30–51 years). The preoperative dysmenorrhea severity score was 8.38 ± 0.70 points (range: 7–9 points); the preoperative menstrual blood loss score was 129.56 ± 13.67 points (range: 105–168 points); 24 patients had varying degrees of anemia, with preoperative hemoglobin concentrations of 101.76 ± 19.17 g/L (range: 72–138 g/L); 31 patients had elevated preoperative CA125 levels, averaging 108.83 ± 128.82 U/mL (range: 11.71–749.90 U/mL); and the average preoperative uterine volume was 205.29 ± 90.44 cm^3^ (range 87.55–445.89 cm^3^).

### 2.2 Treatment methods

#### 2.2.1 Preoperative preparation

Preoperative auxiliary examinations were performed to rule out surgical contraindications. Patients were switched to a liquid diet 2–3 days before surgery for bowel preparation, and skin preparation was completed 1 day prior to surgery.

#### 2.2.2 Anesthesia method and patient position

The patients were placed in the lithotomy position. Following induction of satisfactory general anesthesia with endotracheal intubation, routine disinfection and draping were performed, followed by urethral catheterization.

#### 2.2.3 Surgical procedure

**Figure 1:**
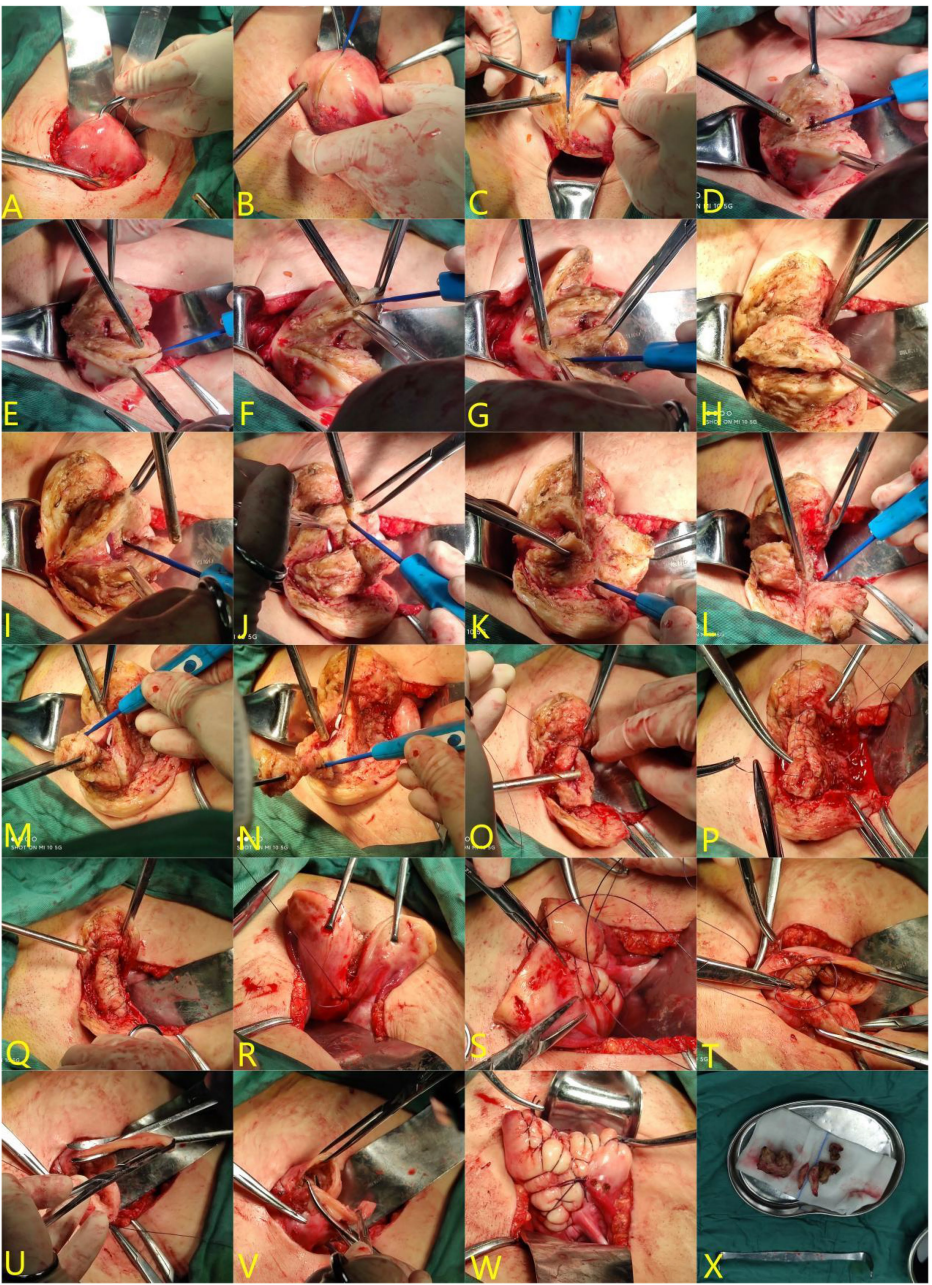
Surgical procedure of modified subtotal resection for adenomyotic lesions. **(A)** Injection of diluted pituitrin; **(B–D)** longitudinal incision of the adenomyotic lesion extending into the uterine cavity; **(E–H)** maximally resecting the lesion (preserving a seromuscular flap of approximately 0.5–1 cm), with the same approach applied to the contralateral lesion; **(I–L)** gradual resection of the lesion to the uterine cavity and partial resection of the cavity to decrease its volume (using the depth of the LNG-IUS as a reference); **(M,N)** the same procedure applied to the contralateral side; **(O–Q)** continuous suturing to reshape the uterine cavity; **(R–T)** “Baseball suture technique” for suturing the uterine seromuscular layer, ensuring proper alignment at the base to eliminate “dead space”; **(U,V)** approximation and trimming of excess uterine seromuscular tissue; **(W)** the reshaped uterus post-repair suturing; **(X)** intraoperative specimen of the resected adenomyotic lesion.

Note: It needs to be emphasized that, due to the absence of a clear histological boundary between adenomyotic lesions and normal myometrium, the so-called “macroscopically complete excision” refers to an intraoperative assessment based on the surgeon’s visual inspection and palpation. This study defines this step as “subtotal lesion resection”, acknowledging the objective possibility of microscopic residual lesions. This limitation forms the theoretical basis for postoperative continuation of combined LNG-IUS therapy, providing long-term medical treatment and management of residual lesions.

A midline vertical incision was made in the lower abdomen, or a previous transverse surgical scar, when present, was used as the approach. The length of the incision was determined based on the size of the uterus and the position of the uterine fundus. After entering the abdominal cavity layer by layer, the pelvic and abdominal cavities were explored for any other abnormalities. The uterus was then pulled out of the pelvic cavity, and the location of the lesion was determined by palpation. A diluted solution of pituitrin was injected into the normal myometrium near the lesion tissue (with close monitoring of blood pressure fluctuations) (Figure 1A). An electrosurgical knife was used to longitudinally incise the adenomyotic lesion, bisecting it to the level of reaching the uterine cavity (Figures 1B–D). Subsequently, the lesion was resected from the seromuscular layer of both separated uterine myometrial flaps, preserving a flap thickness of approximately 5–10 mm for suturing (Figures 1E–H). After the lesions in both myometrial layers had been resected, the peripheral lesions around the uterine cavity were thoroughly excised to minimize postoperative recurrence, with the uterine cavity serving as the central reference point (Figures 1I–N). Ultimately, the endometrium and approximately 5 mm of the adjacent myometrium were preserved as the “uterine center.” After the lesions had been removed as completely as possible, a portion of the uterine cavity was resected and reshaped. An LNG-IUS was placed intraoperatively (or had been placed preoperatively), and the uterine cavity was reshaped to a depth of approximately 7–8 cm using the LNG-IUS as a reference. After all macroscopically visible adenomyotic lesions had been resected, the uterine cavity was continuously sutured (Figures 1O–Q). A “baseball suture technique” was used to suture the bilateral seromuscular layers, ensuring proper alignment of the base to eliminate “dead space” (Figures 1R–T). The bilateral seromuscular layers were approximated, with any excess length trimmed (Figures 1U,V), and suturing was continued to complete the uterine reshaping (Figure 1W). Upon completion of uterine remodeling, the pelvic and abdominal cavities were copiously irrigated with warm saline to minimize the risk of endometrial implantation. The abdomen was closed in layers, with a pelvic drain placed if necessary.

#### 2.2.4 Postoperative management

No specific postoperative intervention was required for the patients. After discharge, GnRH-a injection therapy was initiated on the first day of menstruation (if menstruation did not resume after LNG-IUS insertion, the first injection may be administered based on the previous menstrual cycle). Depending on factors such as the patient’s age and disease condition, 3–6 injections were administered at 28-day intervals, with regular follow-up.

##### 2.2.4.1 Outcome measures

Preoperative, intraoperative, and postoperative measures were recorded to evaluate treatment efficacy. The outcome measures included the following: (1) surgery duration and intraoperative blood loss; (2) the dysmenorrhea severity score was assessed before surgery, at the first menstrual recurrence following completion of the course of GnRH-a injections, and at 12 months postoperatively. The dysmenorrhea severity score was assessed using the visual analog scale (VAS) (11); (3) the menstrual blood loss score was assessed before surgery, at the first menstrual recurrence after completing the course of GnRH-a injections, and at 12 months postoperatively. The menstrual blood loss score was assessed using the Pictorial Blood Loss Assessment Chart (PBAC) (12); (4) hemoglobin concentration: was measured preoperatively, at 3 months, 6 months, and 12 months postoperatively; (5) CA125 level was measured preoperatively, at 3 months, 6 months, and 12 months postoperatively; (6) ultrasound-estimated uterine volume was assessed preoperatively, at 3 months, 6 months, and 12 months postoperatively; uterine volume was calculated using the ellipsoid volume formula: volume = 0.52 × uterine length (measured by ultrasound) × thickness × width (13); and (7) regular follow-up transvaginal ultrasonography was used to monitor the progression of adenomyosis (AM) lesions and check for any displacement of the LNG-IUS.

### 2.3 Statistical analysis

Statistical analysis was performed using SPSS 26.0 and R 4.5 software. The normality of measurement data was assessed using the Shapiro–Wilk test. Normally distributed data were expressed as mean ± standard deviation ( $\bar{x}$ ± s), whereas non-normally distributed data were presented as median (range). Comparisons of measures between preoperative and postoperative time points were performed using paired *t*-tests if normality assumptions were met; otherwise, Wilcoxon signed-rank tests were used. To quantify the magnitude of efficacy, Cohen’s *d* effect size and its 95% confidence interval (CI) were calculated for changes in primary outcome measures between preoperative and 12-month postoperative assessments. This study is an exploratory case series analysis involving multiple comparisons across several endpoints, and no formal *p*-value adjustment was performed. Therefore, a *p*-value of <0.05 only indicates a threshold for statistical significance, and the interpretation of the results should be based on a comprehensive consideration of effect sizes and clinical relevance. This study is a retrospective case series employing consecutive enrollment without prior sample size estimation.

## 3 Results

### 3.1 Clinical characteristics

A total of 34 patients were included in this study, all of whom underwent surgical treatment according to the protocol. We observed differences in age, body mass index (BMI), reproductive history, symptoms, and prior pharmacotherapy; however, these differences were not statistically significant (Table 1).

**Table 1:** Clinical characteristics of the study patients.

Characteristics	Total (*n* = 34)
Age (years)	40.97 ± 5.13
BMI (kg/m^2^)	24.33 ± 3.74
Parity, *n* (%)	
0	0 (0)
1	21 (61.8)
2	13 (38.2)
Miscarriage, *n* (%)	
0	4 (11.8)
1	6 (17.6)
2	14 (41.2)
≥3	10 (29.4)
Comorbidities, *n* (%)	
Leiomyoma	13 (38.2)
Endometriosis	17 (50.0)
Leiomyoma and endometriosis	5 (14.7)
No leiomyoma and endometriosis	9 (26.5)
Medication treatment failure, *n* (%)	
GnRH agonists	11 (32.4)
LNG-IUS	8 (23.5)
Oral contraceptives	7 (20.6)
GnRH agonist + LNG-IUS	8 (23.5)
Kishi classification, *n* (%)	
Type I (intrinsic)	0 (0.0)
Type II (extrinsic)	6 (17.6)
Type III (focal)	12 (35.3)
Type IV (other)	16 (47.1)

Kishi subtype distribution and outcome stratification: In this study, the distribution of Kishi subtypes was as follows: Type II in 6 cases (17.6%), Type III in 12 cases (35.3%), Type IV in 16 cases (47.1%), and no cases of Type I. Due to the small sample sizes of Type II and Type III (only 6 and 12 cases, respectively) and the consistent improvement trends observed in the primary efficacy outcomes (VAS score, menstrual blood loss score, and uterine volume) at 12 months after surgery across all subgroups, formal statistical comparisons between the subgroups were not conducted. The differential responses to this treatment protocol across Kishi subtypes require further exploration with larger sample sizes.

### 3.2 Surgical outcomes

In this study, all 34 patients successfully completed the surgery. The surgery duration was 140.15 ± 33.34 min (range: 75–230 min), with a median intraoperative blood loss of 100 mL (range: 20–500 mL). No postoperative surgical complications were observed.

#### 3.2.1 Postoperative improvement of dysmenorrhea severity

**Figure 2:**
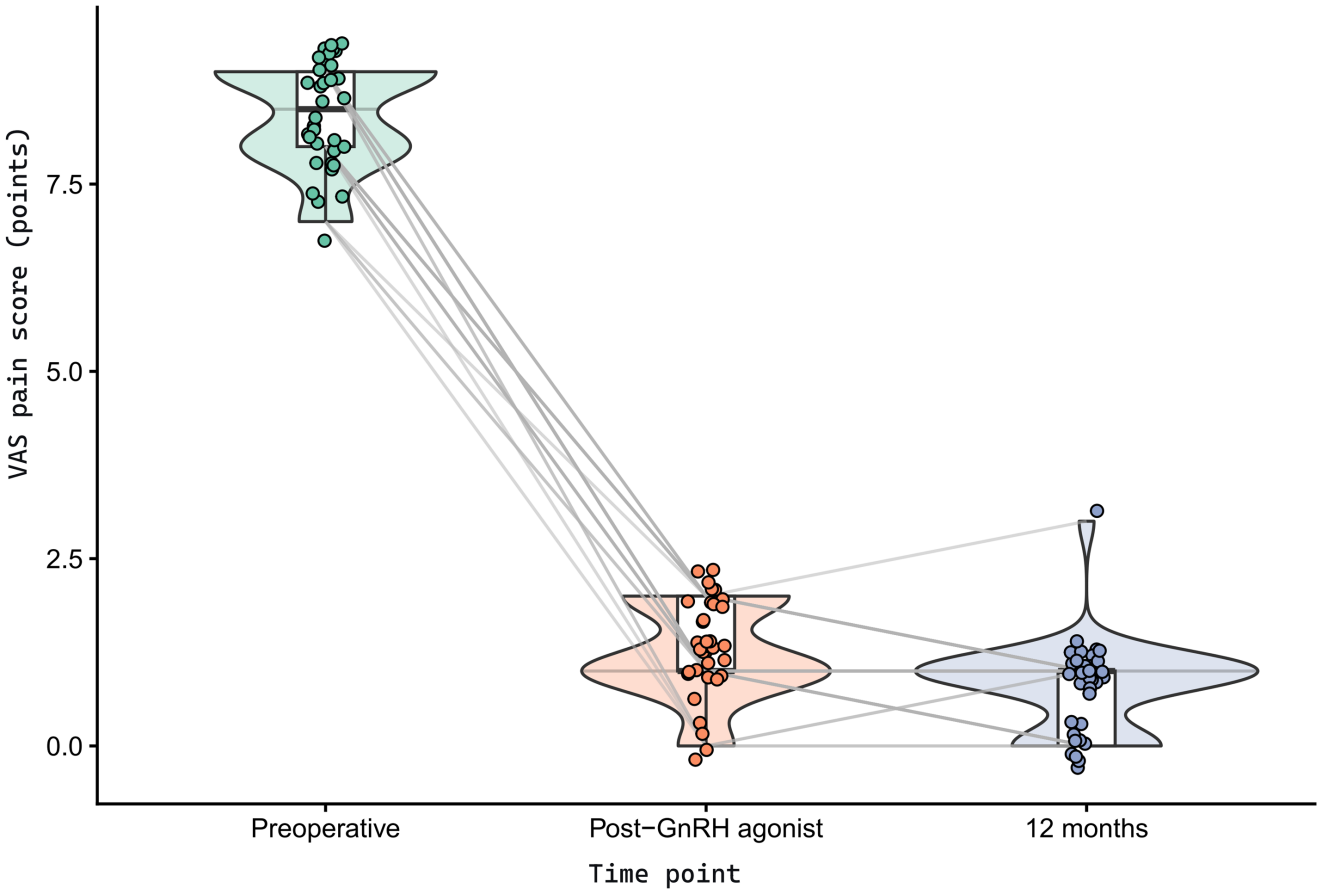
Changes in VAS pain scores.

The visual analog scale (VAS) scoring method was used to assess the severity of dysmenorrhea preoperatively, following completion of the course of the GnRH-a injections and following the return of menstruation, and at 12 months postoperatively. The preoperative VAS score was 8.38 ± 0.70 points (range: 7–9 points). Following the completion of the GnRH-a injection course and the return of menstruation, the VAS score was 1.24 ± 0.65 points (range: 0–2 points). At 12 months postoperatively, the VAS score was 0.76 ± 0.61 points (range: 0–3 points). Compared with preoperative scores, significant improvements were observed (*p* < 0.001), as shown in Figure 2.

#### 3.2.2 Postoperative improvement of menstrual blood loss

**Figure 3:**
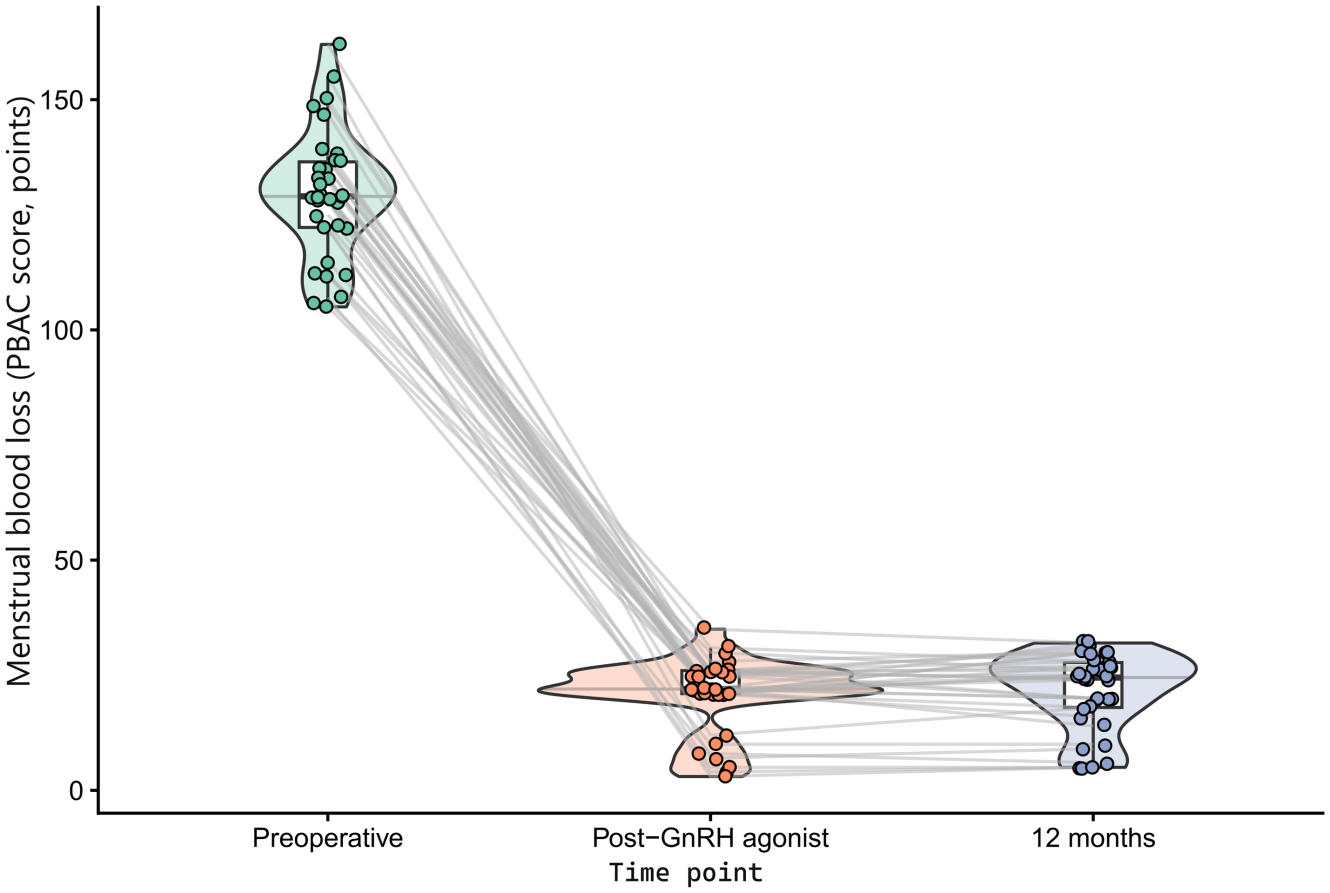
Changes in menstrual volume scores.

The Pictorial Blood Loss Assessment Chart (PBAC) scoring method was used to evaluate menstrual blood loss preoperatively, after sequential GnRH-a-induced menstruation, and at 12 months postoperatively. Preoperative scores were 129.56 ± 13.67 points (range: 105–168 points). Following GnRH-a sequential menstruation, the score decreased to 20.82 ± 7.94 points (range: 3–31 points), and at 12 months postoperatively, it was 21.62 ± 8.38 points (range: 5–32 points). Both postoperative assessments showed significant improvements compared to preoperative scores, with a *p-*value of <0.001, as illustrated in Figure 3.

#### 3.2.3 Postoperative improvement of hemoglobin concentration

**Figure 4:**
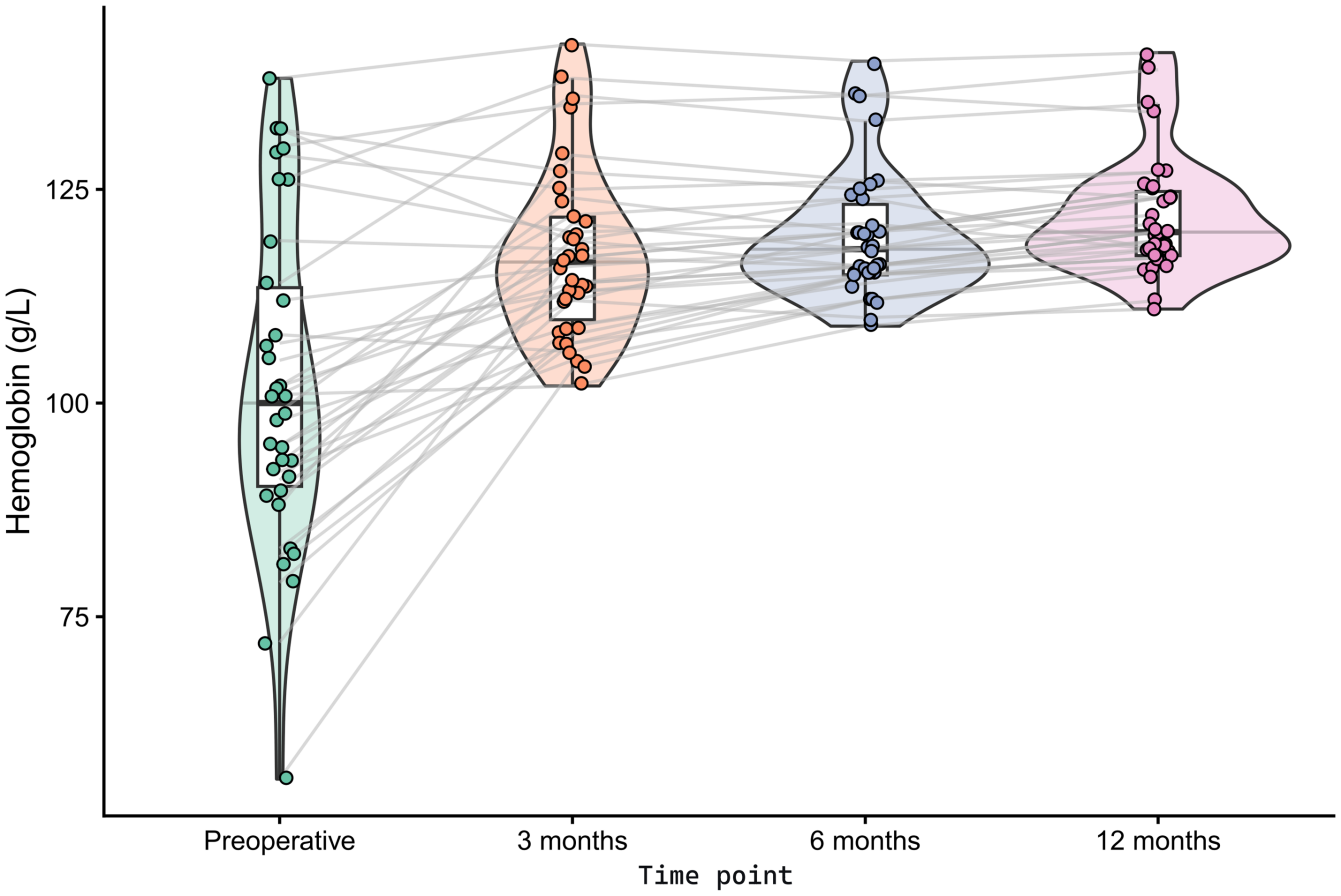
Changes in hemoglobin levels.

The preoperative hemoglobin concentration was 101.76 ± 19.17 g/L (range: 72–138 g/L). At 3 months postoperatively, the hemoglobin concentration was 117.38 ± 10.06 g/L (range: 102–142 g/L); at 6 months, it was 119.68 ± 7.53 g/L (range: 109–140 g/L); and at 12 months, it was 121.91 ± 7.00 g/L (range: 111–141 g/L). Each postoperative measurement showed a significant improvement compared to the preoperative level, with a *p-*value of <0.001, as illustrated in Figure 4.

#### 3.2.4 Postoperative changes in CA125 levels

**Figure 5:**
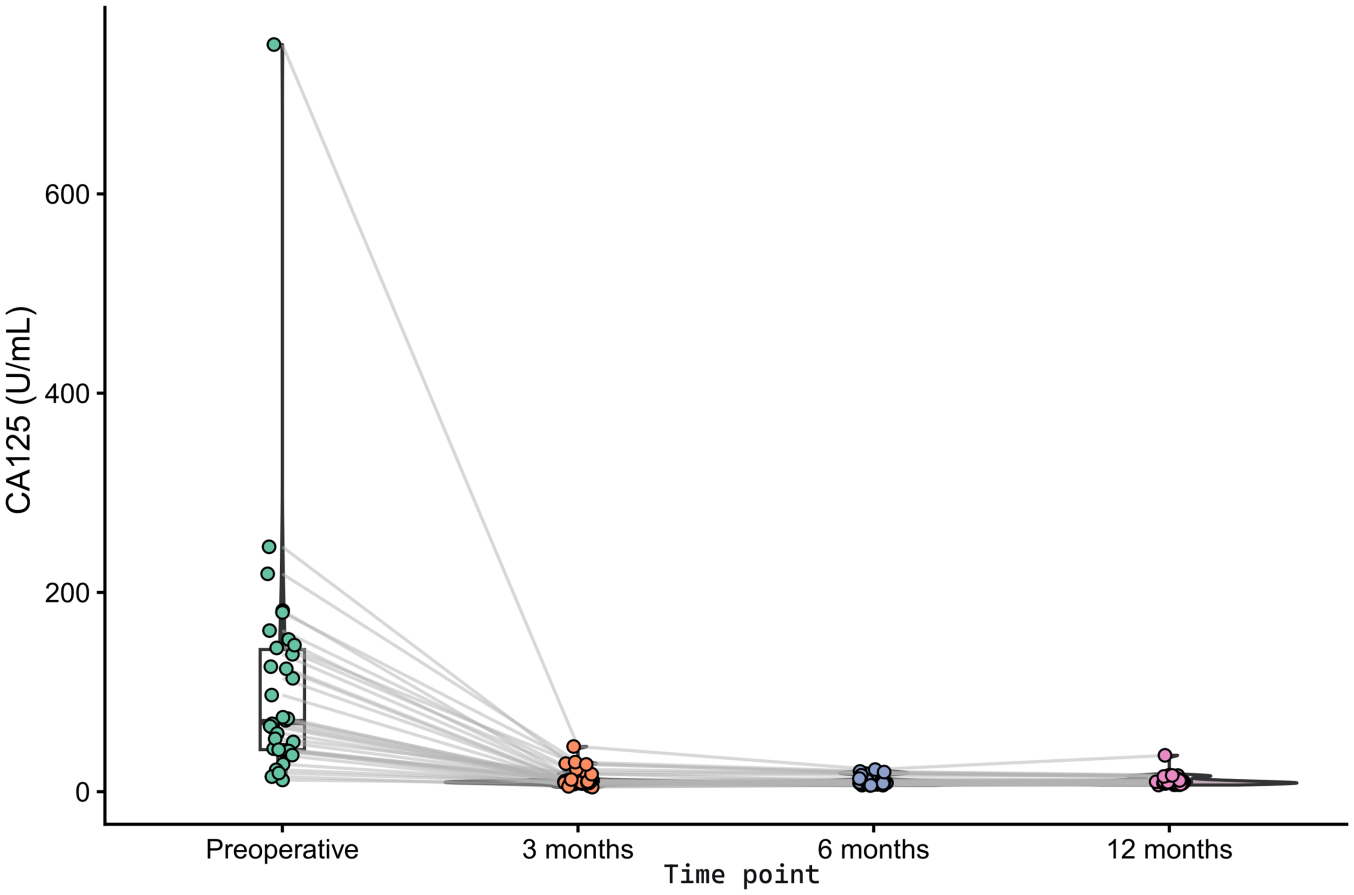
Changes in CA125 levels.

The preoperative CA125 level was 108.83 ± 128.82 U/mL (range: 11.71–749.90 U/mL). At 3 months postoperatively, it decreased to 13.36 ± 9.01 U/mL (range: 4.58–45.36 U/mL); at 6 months, it was 10.97 ± 4.55 U/mL (range: 6.25–22.24 U/mL); and at 12 months, it was 11.06 ± 5.37 U/mL (range: 6.66–36.52 U/mL). Each postoperative measurement showed a significant improvement compared to the preoperative level, with a *p-*value of <0.001, as illustrated in Figure 5.

#### 3.2.5 Postoperative changes in ultrasound-estimated uterine volume

**Figure 6:**
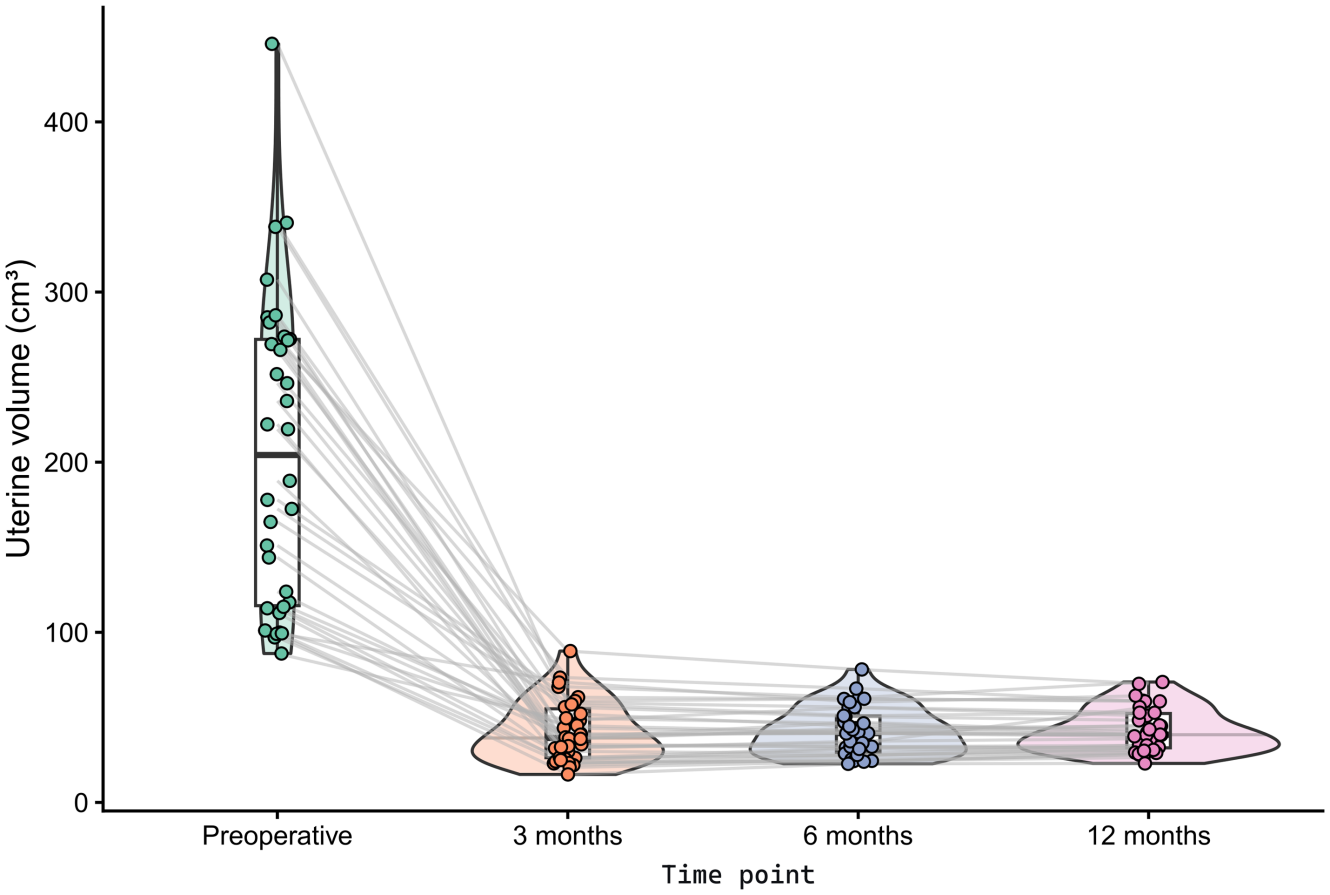
Changes in uterine volume.

The uterine volume changes of patients before surgery and at 3 months, 6 months, and 12 months postoperatively were estimated using the formula: volume = 0.52 × uterine length (measured through transvaginal ultrasonography) × thickness × width. The preoperative average uterine volume estimated through transvaginal ultrasonography was 205.29 ± 90.44 cm^3^ (range: 87.55–445.89 cm^3^). At 3 months postoperatively, the estimated average uterine volume was 41.78 ± 17.79 cm^3^ (range: 16.47–88.97 cm^3^). At 6 months postoperatively, it was 41.80 ± 14.32 cm^3^ (range: 22.71–78.17 cm^3^), and at 12 months postoperatively, it was 42.42 ± 12.97 cm^3^ (range: 22.93–70.79 cm^3^). Significant improvements were observed compared to preoperative values, with a *p-*value of <0.001, as shown in Figure 6.

### 3.3 The median follow-up duration was 12 months (range: 12–15 months)

All 34 patients completed the 12-month postoperative follow-up evaluation. During the postoperative follow-up period, apart from one case of LNG-IUS downward displacement, no other common LNG-IUS-related adverse reactions, such as spotting, weight gain, or breast tenderness, were observed. No patient had the LNG-IUS removed prematurely due to adverse reactions. One patient experienced a mild elevation in CA125 accompanied by an increase in dysmenorrhea severity score to 3 points, with no significant increase in menstrual blood loss observed at that time, and no progression of lesions was noted in this patient (see Table 2).

**Table 2:** Paired comparison of outcome variables between preoperative baseline and the 12-month postoperative follow-up in 34 patients with severe adenomyosis.

Variable	Preoperative	12 months	Mean change	95% CI of change	Cohen‘s *d*	95% CI of d	*p*-value
VAS score (points)	8.38 ± 0.70	0.76 ± 0.61	−7.62	(−7.94, −7.30)	−8.27	(−10.26, −6.27)	<0.001
Menstrual blood loss (PBAC)	129.56 ± 13.67	21.62 ± 8.38	−107.94	(−113.30, −102.58)	−7.02	(−8.73, −5.32)	<0.001
Hemoglobin (g/L)	101.76 ± 19.17	121.91 ± 7.00	20.15	(14.39, 25.90)	1.22	(0.78, 1.66)	<0.001
CA125 (U/mL)	108.83 ± 128.82	11.06 ± 5.37	−97.77	(−141.06, −54.49)	−0.79	(−1.17, −0.40)	<0.001
Uterine volume (cm^3^)	205.29 ± 90.44	42.42 ± 12.97	−162.87	(−193.40, −132.34)	−1.86	(−2.42, −1.31)	<0.001

Values are mean ± SD unless otherwise specified. CI, confidence interval; PBAC, Pictorial Blood Loss Assessment Chart.

## 4 Discussion

In 2012, Kishi et al. (8) classified adenomyosis into four subtypes based on MRI imaging characteristics: Subtype I (intrinsic type), in which lesions infiltrate and distribute in the inner layer of the uterus without affecting the outer layer; Subtype II (extrinsic type), in which lesions infiltrate and distribute in the outer layer of the uterus without affecting the inner layer; Subtype III (focal type), including adenomyoma and cystic adenomyosis; and Subtype IV (other types) (8). In recent years, additional MRI classifications for adenomyosis have been proposed, including the Chapron et al. (5) classification in 2017 and the Bazot and Daraï (14) classification in 2018. In this study, the Kishi classification was used to describe the anatomical location and infiltration pattern of lesions to guide surgical resection planning, whereas the FIGO grading (myometrial involvement >50%) was used as the criterion for defining “severe” adenomyosis and determining eligibility for this study. The two systems are complementary: the Kishi classification guides surgical resection scope, and the FIGO grading defines patient inclusion criteria. Since the lesions of Subtype I disease are distributed in the inner layer of the uterus, the LNG-IUS can directly deliver its therapeutic effects to the lesion site, making it an effective treatment. In contrast, for other subtypes, the drug cannot exert a direct local effect on the lesion surface, resulting in suboptimal therapeutic outcomes, thereby making surgical intervention more suitable. We believe that the Kishi classification is more concise and clear, offering an intuitive overview of adenomyosis to guide clinical treatment. Reports have suggested that MRI has an estimated sensitivity, specificity, positive predictive value, and negative predictive value of 77.5, 92.5, 83.8, and 89.2%, respectively, for diagnosing adenomyosis (15). However, due to the high cost of MRI, some patients are unable to undergo it as an auxiliary diagnostic method. With the advancement of transvaginal ultrasonography in recent years, it has been increasingly recognized that this modality also holds certain diagnostic value for adenomyosis (16–18). Studies have indicated that its specificity and sensitivity for diagnosing adenomyosis are estimated at 84 and 64%, respectively (19). Transvaginal ultrasonography is more economically accessible to patients, and the Kishi classification, due to its simplicity, can be effectively applied in transvaginal ultrasound diagnostics. Therefore, this treatment protocol also permits the use of transvaginal ultrasound as an auxiliary preoperative diagnostic examination.

The LNG-IUS continuously releases a controlled dose of levonorgestrel into the uterine cavity via a sustained-release mechanism, achieving therapeutic effects by infiltrating the myometrium. Only a minimal amount enters systemic circulation. The high local drug concentration directly reduces estrogen receptor activity in lesions, blocks estrogen effects, and promotes lesion differentiation and atrophy (20). However, as an intrauterine device, the LNG-IUS is associated with a higher expulsion rate when placed in patients with heavy menstrual bleeding or a larger uterine cavity. In patients with adenomyosis, the uterine volume often increases, frequently accompanied by an enlarged uterine cavity and increased menstrual bleeding. Prior to LNG-IUS treatment for adenomyosis, GnRH-a is often administered for 3 months to reduce the expulsion rate (20). There are numerous reports on the use of LNG-IUS for treating adenomyosis, and its efficacy has been well documented (21–24). However, a 5-year prospective study has revealed that, even with GnRH-a pretreatment, the expulsion rate of LNG-IUS in adenomyosis patients remains as high as 21.8% (25). To minimize the expulsion rate of LNG-IUS in this protocol and improve therapeutic outcomes, patients were placed in the lithotomy position during surgery. The adenomyotic lesion was incised to reach the uterine cavity, following which Mirena was placed and the uterine cavity was reshaped based on its depth, thereby lowering the expulsion risk related to uterine cavity enlargement. Postoperatively, patients continue to receive 3 months of GnRH-a injection therapy. We believe that the combined administration of GnRH-a and LNG-IUS can further reduce residual lesions and lower the likelihood of LNG-IUS expulsion. Based on our follow-up data, this approach has shown preliminary effectiveness. This empirical 3-month course of GnRH-a therapy has been documented in previous studies (26).

The boundary between adenomyotic lesions and the surrounding normal myometrium is unclear. If residual lesions remain after surgical excision, they may gradually enlarge postoperatively due to the cyclical fluctuations in the patient’s hormonal levels. Therefore, postoperative management (standardized treatment and follow-up) is also critically important (7). However, the efficacy of drug therapy for adenomyosis is currently unsatisfactory, and the probability of postoperative recurrence is closely related to the size of residual lesions during surgery. In patients who desire future pregnancies post-surgery, the need to reduce the risk of uterine rupture during pregnancy inevitably interferes with the complete resection of lesions, which may result in residual lesion persistence. In patients without future pregnancy intentions who wish to preserve their uterus after failed medical treatment, the challenge lies in maximizing lesion removal during surgery, even when aiming for “complete eradication,” which warrants careful consideration. Adenomyosis shares similar biological characteristics with endometrial cancer (27). Therefore, we introduced the concept of “radical resection” into the treatment of adenomyosis and made certain improvements to the “double-flap” technique for adenomyosis excision. This surgical approach involves the extensive resection of myometrial lesions in Kishi classification Subtypes II, III, and IV of adenomyosis. Only 5–10 mm of the myometrial layer near the serosal side is preserved in the bilateral uterine myometrial flaps, while approximately 5 mm of the myometrial layer adjacent to the endometrium is retained as the “uterine center.” This method aims to reshape the uterus and achieve the goal of removing as much lesion tissue as possible. If residual lesions remain in the reshaped uterus, their proximity to the endometrium can be considered as Subtype I in the Kishi classification. The intraoperative placement of LNG-IUS in this treatment plan allows for the continuous release of levonorgestrel into the uterine cavity, targeting the lesions. For adenomyotic lesions close to the uterine cavity, LNG-IUS exhibits certain therapeutic effects (21–24). It is worth emphasizing that the goal of this surgical approach is not pathological “complete resection” (R0 resection); however, the goal is the maximal reduction of lesion burden through surgery, transforming the anatomically complex subtypes (Kishi classification subtypes II–IV), which originally responded poorly to medical therapy alone, into a “functionally converted” state in which the lesions are adjacent to the uterine cavity, thereby facilitating progesterone penetration, akin to the “Kishi classification subtype I” condition. Postoperatively, leaving the LNG-IUS in place provides a long-term management strategy precisely for these potential residual lesions. Thus, “radical excision” (surgery) and “sustained suppression” (medication) form the synergistic core of this protocol.

This study observed a noteworthy phenomenon: All primary efficacy indicators (VAS dysmenorrhea severity score, menstrual blood loss, hemoglobin concentration, CA125, and uterine volume) showed significant improvements compared to preoperative levels at the first follow-up after completing the GnRH-a treatment course. Moreover, this degree of improvement was consistently maintained during the 12-month postoperative follow-up period, with no obvious symptom rebound or disease recurrence. We hypothesize that this dynamic pattern of “rapid onset and sustained stability” reflects the temporal synergy of the three components in this combined regimen: (1) Surgical phase: The subtotal resection of lesions immediately removes the majority of pathological tissue, serving as the structural foundation for symptom relief; (2) consolidation phase (1–3 months postoperatively): GnRH-a induces a transient hypoestrogenic state by suppressing the pituitary-ovarian axis, leading to rapid atrophy of residual lesions and ectopic endometrium, while potentially reducing the risk of early LNG-IUS displacement by minimizing menstrual recurrence; and (3) maintenance phase (3–12 months postoperatively and beyond): After the effects of GnRH-a subside, the LNG-IUS leverages its sustained high-concentration local release of levonorgestrel within the uterine cavity to chronically suppress the proliferative activity of residual lesions, thereby maintaining a therapeutic plateau. This model provides a preliminary pharmacodynamic rationale for the treatment sequence of “debulking surgery + short-term GnRH-a + long-term LNG-IUS”.

Based on the Kishi classification concept, the improvements in this protocol offer the following advantages: (1) The surgical modification aims to transform Subtypes II, III, and IV adenomyosis into “Subtype I” adenomyosis through surgery, allowing LNG-IUS to serve as a long-term treatment; (2) the introduction of the “radical resection” concept maximizes the removal of myometrial lesions, laying the foundation for reduced recurrence in subsequent treatments; (3) intraoperative placement of LNG-IUS provides uterine cavity guidance, reshaping the cavity and enhancing compliance with maintenance therapy; (4) this protocol uses an open abdominal approach, enabling the tactile assessment of lesions by the surgeon for more thorough excision; and (5) the “baseball suture technique” ensures tighter closure, minimizing hematoma formation. The limitations include the following: (1) The empirical use of 3-month GnRH-a therapy postoperatively lacks robust evidence regarding its necessity or optimal duration, despite reports supporting its efficacy in combination with LNG-IUS for adenomyosis, and (2) there is a potential risk of iatrogenic endometriosis implantation during the procedure. To mitigate this, we emphasize that the uterine cavity should be closed as promptly as possible to minimize exposure time. Additionally, copious irrigation of the pelvic and abdominal cavities with warm saline should be performed after uterine remodeling to reduce the potential dissemination of residual endometrial cells.

Additionally, in this study, cases with uterine volumes at the lower range limit (87.55 cm^3^), although not large in absolute terms, exhibited preoperative ultrasound findings of diffuse adenomyotic lesions involving the entire myometrial wall, with the proportion of affected muscle layers far exceeding 50%. These patients also suffered from long-standing severe dysmenorrhea (VAS 7–9) and menorrhagia, which were refractory to multiple conservative treatments. Therefore, the primary rationale for proceeding with surgery was the relative extent of lesion infiltration and the resulting intractable symptoms rather than the absolute uterine volume. This also indirectly suggests that this surgical approach is more suitable for severe cases characterized by diffuse lesions, pronounced symptoms, and poor response to pharmacological treatments.

This study has the following main limitations. First, the study design was a single-center, retrospective case series without parallel control groups—such as an LNG-IUS-only treatment group, a GnRH-a-only treatment group, or a hysterectomy group. This makes it impossible to fully attribute the observed efficacy to any specific component of the combined regimen, nor can the study rule out placebo effects or natural fluctuations in disease activity. Therefore, this study serves solely as an exploratory investigation of efficacy, and causal conclusions must await prospective randomized controlled trials. Second, the sample size was small (*n* = 34), and patients were enrolled consecutively without *a priori* sample size estimation, which may have introduced selection bias. Moreover, the small sample size limited statistical power, making it difficult to detect subtle differences in treatment effects (increased risk of Type II error) and precluded stratified analyses across Kishi subtypes to identify subgroups most likely to benefit. Third, the 12-month follow-up period is insufficient for evaluating long-term outcomes of adenomyosis—a chronic disease with high recurrence rates—particularly regarding symptom recurrence rates at 2–5 years post-surgery, long-term LNG-IUS retention rates, and the long-term uterine rupture risk. Fourth, all surgeries were performed by experienced gynecologists, and the generalizability of the results across different surgeons and institution levels remains to be validated. Fifth, this study involved comparisons of multiple outcome measures and multiple time points without adjusting the *p*-values for multiplicity, which may have increased the probability of Type I errors (false positives). Sixth, since this was a retrospective study and some patients underwent ultrasound rather than MRI, precise junctional zone thickness measurements were not systematically available. This should be prospectively collected in future studies to refine patient selection.

## Data Availability

The data analyzed in this study is subject to the following licenses/restrictions: Internal Statistics of Hospitals. Requests to access these datasets should be directed to Zhenyue Qin, qinzy0622@163.com.
